# Biopsies of the normal-appearing urothelium in primary bladder cancer

**DOI:** 10.4103/0974-7796.65115

**Published:** 2010

**Authors:** Davor Librenjak, Zana Saratlija Novakovic, Marijan Situm, Kazimir Milostic, Mario Duvnjak

**Affiliations:** Department of Urology, Clinical Hospital Center, School of Medicine, Soltanska 1, 21000 Split, Croatia

**Keywords:** Normal-appearing urothelium, bladder biopsy, bladder cancer, TUR

## Abstract

**Aim:**

The aim of the study was to determine the incidence of "positive" findings in biopsies of the normal-appearing urothelium near primary cancer and their influence on therapeutic decisions.

**Materials and Methods:**

Between January 2001 and October 2008, in 230 patients with primary bladder cancer during initial resection of tumor, we also performed random biopsy of surrounding normal-appearing urothelium. We analyzed retrospectively the number and type of positive biopsy findings and their impact on further treatment.

**Results:**

There were 40% of patients (92/230) whose normal-appearing urothelium biopsy revealed pathological findings such as tumor tissue, Tis, and dysplasia. In 24.4% of patients, the stage of the primary tumor was Ta (32/131), in 50% it was T1 stage (30/61), and in 79% T2 stage (30/38). When we assessed the grade of malignancy, we found 18% of biopsies with G1 tumors (16/88), 33% with G2 tumors (19/59), and 69% with G3 tumors (57/83). Tumor tissue that was found in the normal-appearing urothelium in biopsy specimens in 13% of patients was in stage Ta (17/131), in 16% it was T1 stage (10/61), and in 39% of patients, the tumor was in T2 stage (15/38). Pathological findings of random biopsies were crucial in changing therapeutical decisions in 4.6% (9/192) of patients.

**Conclusion:**

Biopsy of the normal-appearing urothelial tissue is easy to perform and may help in identifying patients with high risk of disease progression and recurrence. Based on our results and results from the literature we recommend this simple tool as part of the routine management during transurethral resection of primary bladder cancer.

## INTRODUCTION

Transurethral resection (TUR) is the initial diagnostic and therapeutic procedure in the treatment of the urinary bladder tumors, and the quality of its performance significantly determines further course of the illness. With TUR, a urologist removes all macroscopically visible lesions of urothelium and gets adequate pathohistological material for analysis. Analyzing biopsy tissue specimen, a pathologist determines the type of tumor, and estimates the grade of malignancy and the depth of tumor infiltration in the bladder wall (stage). Approximately 75–80% tumors at initial presentation are muscle noninvasive (former name superficial), and in 20–25% of cases are muscle invasive bladder tumors.[1]

Muscle noninvasive tumors are mucosa-confined tumors (Ta), tumors which affect lamina propria (T1), and tumor *in situ* (Tis). Recurrence of up to 30% of solitary papillary tumors is expected during the first year after TUR, as well as up to 90% of multiple tumors with a higher grade affecting lamina propria.[2] The basic causes of tumor recurrence are implantation during primary resection (large and multiple tumors), undiscovered residual tumor (later manifested as a recurrence), and the existence of macroscopically invisible premalignant and malignant lesions of urothelium during the primary resection. The existence of these lesions can be detected by taking biopsy from apparently normal mucosa in the vicinity of the tumor during the initial TUR. The significance of random bladder biopsies in patients with superficial bladder cancer is still controversial. Today, there is no consensus about the usefulness of this procedure and its impact on the further course of the disease.[3-8]

The aim of this study was to determine the incidence of pathological biopsy findings in the normal-appearing urothelium which surrounds the tumor, and their influence on the therapeutic approach in patients with primary bladder tumor.

## MATERIALS AND METHODS

Between January 2001 and October 2008, in 230 patients with primary bladder cancer during initial resection of tumor we took random biopsy specimen from normal-appearing urothelium at edge of the resected tumor. We consider positive findings of biopsy specimen tumor tissue, tumor *in situ* (Tis), and dysplasia. In each patient before surgery, we recorded size, localization, number, and configuration of the tumor. We estimated the size of the tumor by comparing it with the resection loop of a known diameter (6 mm). Tumors smaller than 2 cm were considered small, those between 2 and 5 cm medium size, and above 5 cm large tumors.

### Resection technique

All transurethral resections were performed with an Olympus active resectoscope USE 40, SurgMaster, using monopolar cutting energy of 100 W. After we resected all changes above the mucosa (as separated specimen we resected base of tumors), for a width of a resection loop, we resected the surrounding urothelium without tumors. Then we took biopsy specimens from the normal-appearing mucosa 6 mm away from the resection edge, which was the diameter of a resection loop. The stage of disease was determined according to the TNM classification of malignant tumors from 1997, and the degree of malignancy according to the World Health Organization classification from 1999.[9,10] We obtained information about the features of tumors by searching patient files from archives at the Department of Urology, University Hospital Split, Split, Croatia.

Statistical analysis was performed with Statistica 7.0 software (StatSoft Inc., Tulsa, OH, USA), using the chi-square test. Statistical significance was set at *P*<0.05.

## RESULTS

Among patients there were 176 men, whose mean age was 67±11 years and 54 women aged 68±11 years. Clinical characteristics of primary tumors in relation to prognostic variables are shown in Table 1.

**Table 1:** Features of the primary tumor biopsy

Features	Patient no.	Percentage
Stage		
Ta	131	57
T1	61	26.5
T2	38	16.5
Grade		
I	88	38
II	59	26
III	83	36
Tumor no.		
Solitary	156	68
Multiple	74	32
Size		
Small	85	37
Medium	88	38
Large	57	25

Pathological findings were found in 40% of biopsies in the normal-appearing urothelium (92/230). The number of pathological findings in the normal-appearing urothelium was higher in patients with more advanced stages of the primary tumor [Table 2]. Comparing to stage Ta, the number of positive biopsies in stages T1 and T2 was significantly higher (χ^2^=41, *P*<0.001).

**Table 2:** Pathological findings in biopsies of the normal-appearing urothelium in relation to the primary tumor

Stage of the primary tumor (*n*)	Positive biopsy, *n* (%)	Tumor tissue, *n* (%)	Tis, *n* (%)	Dysplasia, *n* (%)
Ta (131)	32 (24)	17(13)	8 (6)	7 (5)
T1 (61)	30 (50)	10 (17)	11 (18)	9 (15)
T2 (38)	30 (79)	15 (39)	12 (32)	3 (8)

Furthermore, pathological findings were significantly higher in the normal-appearing urothelium of patients whose primary tumor was described with a higher grade (χ^2^=48, *P*<0.001) [Table 3]. Pathological biopsy findings in the normal-appearing urothelium were significantly higher in patients with larger primary tumors (*P*<0.001) [Table 4]. When number of primary tumors was analyzed (solitary versus multiple) there was no statistically significant difference in the number of positive biopsies of the surrounding normal-appearing urothelium (*P*=0.87).

**Table 3:** Pathological findings in biopsies of the normal-appearing urothelium in relation to the malignancy degree of the primary tumor

Grade of the primary tumor (*n*)	Pathological finding in normal urothelium, *n* (%)
I (88)	16 (18)
II (59)	19 (33)
III (83)	57 (69)

**Table 4:** Pathological findings in biopsies of the normal-appearing urothelium in relation to the size of the primary tumor

Size of the primary tumor (cm)	Positive biopsy, *n* (%)
Small (<2)	20/85 (24)
Medium (2–5)	39/88 (44)
Large (>5)	31/57 (54)

Since a positive biopsy finding is important in making therapeutic decisions only in muscle noninvasive tumors, we analyzed the frequency of positive findings in this group of tumors compared to tumor stage and grade [Table 5].

In the group of patients with moderately differentiated TaG2 tumors, we obtained positive biopsy findings in 29% of cases (11/38): in 3 cases were found tumor tissue, in 2 dysplasia, in 1 cystitis cystica, and in 5 cases Tis. Within this group, the biopsy finding was crucial in the therapy making decision in eight respondents (21% of respondents with moderately differentiated Ta tumors, or 4.1% of all subjects with muscle noninvasive cancer). In one case, a T1G3 tumor through biopsy findings (which was spread through the muscle) was classified as muscle invasive. In nine subjects due to random biopsy results (4.6% with muscle noninvasive cancer), choice of therapy was altered.

**Table 5:** Pathological findings in biopsies of normal-appearing urothelium in muscle noninvasive tumors

Classification of a primary tumor	Positive biopsies, *n* (%)
Ta G1	16/84 (19)
Ta G2	11/38 (29)
Ta G3	5/9 (56)
T1 G1	0 (0)
T1 G2	8/23 (35)
T1 G3	22/38 (58)

Taking biopsy specimens from the normal-appearing urothelium 6 mm away from the primary tumor did not prolong the time of resection, neither it was associated with more complications such as bleeding and bladder rupture.

## DISCUSSION

The basic feature of muscle noninvasive bladder cancer is a great tendency to recur, and progression to a lesser extent.[2] The most important cause of recurrence is the character of the disease, which also assumes the existence of visible tumors that were resected and macroscopically invisible premalignant and malignant lesions of urothelium that later manifested as recurrence. According to the data in the literature, currently there is no consensus about the usefulness of the biopsy of the normal-appearing bladder urothelium during the resection of primary muscle noninvasive urothelial cancer. In most of the studies that analyzed this issue, biopsy was taken from random places in bladder, such as both sides of walls, base, floor, posterior wall, dome, prostatic urethra (in males), or a bladder neck (in females). The percentage of positive biopsy findings was between 8% and 23%, and the conclusions on the basis of these results were different.[3-8]

Some authors believe that biopsy of the normal-appearing mucosa during the initial resection does not contribute to the more accurate diagnosis and that it does not significantly affect the decision about treatment. They consider it an unnecessary procedure.[3,4] Others consider it optional for multiple tumors and positive urinary cytology.[5] There are opposing views on whether this procedure should be done routinely at the initial resection, since in the substantial number of patients (4.6%), it has an effect on therapeutic decision.[6,7]

In our study, the biopsy material was taken from the urothelium of normal appearance in the close vicinity of resected tumors, precisely 6 mm away from the primary tumor, as measured with a resection loop. We suspected that just this biopsy material of the "normal-appearing" mucosa surrounding the primary tumor will contain a significant number of microscopic premalignant and malignant lesions. Our approach was provoked by studies of Herr and Vogeli who did a routine secondary resection of the tumor 2-6 weeks after the initial resection.[11,12] In 96 patients with muscle noninvasive tumors, Herr found residual tumor tissue in 75% of cases. In 29% of patients, the tumor was later reclassified in a higher stage.[11] In the group of 215 patients, at secondary resection, Vogeli revealed residual tumors in 37% of patients with Ta tumors and in 43% of patients with T1 tumors. In 9% of cases, the tumor was reclassified to a higher stage.[12] Even in solitary, well-differentiated Ta tumors, residual tumors could be found in 24% of cases.[13]

In our study we have had positive biopsy findings in one third of patients with muscle noninvasive tumors. We propose that the higher percentage of positive findings in our biopsies of the normal-appearing urothelium, in comparison with the data from the literature, is due to the fact that we took biopsies from the lining that closely surrounds the resected tumor base.

In the T1 stage we have, regardless of biopsy findings, indicated BCG immunotherapy because all tumors were moderately or poorly differentiated. In one case we reclassified T1G3 tumors through biopsy findings in muscle-invasive tumors. In Ta stage tumors, because of the positive biopsy findings in eight patients, we decided to administer intravesical BCG immunotherapy (21% of patients with Ta tumors, or 4.6% of all patients with muscle noninvasive tumors). Positive findings at biopsy in 4.6% (9/192) patients with muscle noninvasive tumors affected the decision of the therapeutic approach.

In patients with T1 tumors, the decision on further treatment was less affected by biopsy findings since in all patients we routinely performed second resection and indicated BCG immunotherapy. The value of this procedure is in possible reclassification in early stage of disease based on biopsy findings, which we have done in one case.

The percentage of positive biopsy findings correlated with tumor stage, malignancy grade, and tumor size, while the number of tumors did not affect the frequency of positive biopsy findings. These results were expected and verify that tumor stage, malignancy grade, and size of tumors are strong predictors of clinical course of disease.

Tis was noted in 10.4% cases and dysplasia in 7.8% cases of muscle noninvasive tumors. According to data from the literature, concomitant Tis is a bad prognostic sign and is associated with a higher probability of progression.[14-16] Some authors consider it to be a precursor of muscle-invasive urothelial carcinoma.[17] Dysplasia associated with urothelial carcinoma is also associated with greater probability of recurrence and progression.[18,19]

Although the incidence of positive biopsy findings is relatively homogenous among different authors (8–23%), they draw different conclusions from these results. The authors who propose in their publications that the routine biopsy of the normal-appearing mucosa is unnecessary showed that a positive biopsy finding is not an independent predictor of recurrence and progression of disease, and that a small number of patients are affected by a change in the therapeutic approach.[3,4] Proponents of the routine biopsy argue that in 6-7% of patients, based on biopsy findings, the therapeutic approach is changed. According to them, that number is significant and justifies routine biopsy of the normal-appearing mucosa.[6-8]

Based on the results of our study, we believe that the routine random biopsy of normal-appearing mucosa that closely surrounds tumors contributes to a more precise insight into the status of urothelium. We believe that 4.6% of patients, in whom a positive biopsy finding is crucial in making therapeutic decisions, is significant since BCG immunotherapy indicated according biopsy finding has proven efficacy in preventing recurrence and progression of muscle noninvasive bladder cancer.[20,21]

In our study, the clinical course of disease in patients with biopsy of the normal-appearing urothelium was not further followed and compared to group of patients in which this biopsy was not done. We think that the evaluation of the impact of normal-appearing mucosa biopsy in the patient's clinical course over a longer time is delicate, since the dominant impact on the evolution of the disease has strong prognostic variables such as stage, grade, and tumor size. Therefore, we believe that it is not realistic to expect biopsy findings of the normal-appearing urothelium to be an independent prognostic factor for recurrence and progression rate through a longer period of follow-up.

Taking the biopsy material from the normal-appearing mucosa that surrounds tumors, in fact, we extend the resection. In that way, the quality of resection raises and we acquire more accurate insight into the status of the urothelium. If we take into account the frequency of muscle noninvasive tumors in a significant number of patients, routine biopsy will mean changing the treatment plan. Such resection can reduce the percentage of residual tumors at the resection site and consequently the number of orthoptic recurrence. Taking the biopsy material does not extend the time of endoscopic procedure and is not associated with the occurrence of complications such as bladder rupture.

Taking the biopsy material from the normal-appearing mucosa near the resection edge is a safe procedure which allows, based on biopsy findings, a change in the therapeutic approach for a significant number of patients.

## References

[1] Freeman JA, Esrig D, Stein JP, Simoneau AR, Skinner EC, Chen SC (1995). Radical cystectomyfor high risk patients with superficial bladder cancer in the era of orthoptic urinary reconstruction. Cancer.

[2] Herr HW, Laudone VP, Whitmore WF (1987). An overwiew of intravesical therapy for superficial bladder tumors. J Urol.

[3] Kiemeney LA, Witjes JA, Heijbroek RP, Koper NP, Verbeek AL, Debruyne FM (1994). Should random urothelial biopses be taken from patients with primary superficial bladder cancer? A decision analysis. Br J Urol.

[4] van der Meijden A, Oosterlinck W, Brausi M, Kurth KH, Sylvester R, de Balincourt C (1999). Significance of bladder biopsies in Ta, T1 bladder tumors: a report from the EORTC Genito-Urinary Tract cancer Cooperative Group. EORTC_GU Group Superficial Bladder Committee. Eur Urol.

[5] Fujimoto N, Harada S, Terado M, Sato H, Matsumoto T (2003). Multiple biopsies of normal-looking urothelium in patients with superficial bladder cancer: Are they necessary. Int J Urol.

[6] May F, Treiber U, Hartung R, Schwaibold H (2003). Significance of random bladder biopsies in superficial bladder cancer. Eur Urol.

[7] Kiemeney LA, Witjes JA, Heijbroek RP, Debruyne FM, Verbeek AL (1994). Dysplasia in normal-looking urothelium increases the risk of tumor progression in primary superficial bladder cancer. Eur J Cancer.

[8] Mufti R, Singh M (1992). Value of random mucosal biopsies in the management of superficial bladder cancer. Eur Urol.

[9] Sobin DH, Witteking C (1977). UICC: classification of malignant tumours.

[10] Epstein J, Amin M, Reuter V, Mostofi F (1998). The Bladder Consensus Conference Committee. The World Health Organization/International Society of Urological Pathology consensus classification of urothelial (transitional cell) neoplasms of the urinary bladder. Am J Surg Pathol.

[11] Herr HW (1999). The value of a second transurethral resection in evaluating patients with bladder tumors. J Urol.

[12] Vogeli TA, Grimm M, Ackermann R (1998). Prospective study for quality control of TUR of bladder tumors by routine second TUR (reTUR). J Urol.

[13] Kőhrann KU, Woeste M, Kappes J (1994). The value of transurethral resection for superficial bladder cancer. Akt Urol.

[14] Bostwick DG, Ramnani D, Chang L (1999). Diagnosis and grading of bladder cancer and associated lesions. Urol Clin North Am.

[15] Orozco RE, Martin AA, Murphy WM (1994). Carcinoma *in situ* of the urinary bladder. Cancer.

[16] Cheng L, Cheville JC, Neumann RM, Leibovich BC, Egan KS, Spotts BE (1999). Survival in patients with carcinoma *in situ* of the urinary bladder. Cancer.

[17] Spruck CH, Ohneseit PF, Gonzalez-Zulueta M, Esrig D, Miyao N, Tsai YC (1994). Two molecular pathways to transitional cell carcinoma of the bladder. Cancer Res.

[18] Smith G, Elton RA, Beynon LL, Newsam JE, Chisholm GD, Hargreave TB (1983). Prognostic significance of biopsy results of normal-looking mucosa in cases of superficial bladder cancer. Br J Urol.

[19] Heney NM, Ahmed S, Flanagan MJ, Frable W, Corder MP, Hafermann MD (1983). Superficial bladder cancer: progression and recurrence. J Urol.

[20] Lamm DL, Blumenstein BA, Crissman JD, Montie JE, Gottesman JE, Lowe BA (2000). Maintenance bacillus Calmette-Guerin immunotherapy for recurrent Ta, T1 and carcinoma *in situ* transitional cell carcinoma of the bladder: a randomized Southwest Oncology Group Study. J Urol.

[21] Sylvester RJ, Van der Meijden PM, Lamm DL (2002). Intravesical bacillus Clamette-Guerin reduces the risk of progression in patients with superficial bladder cancer: A meta-analysis of the published results of randomized clinical trials. J Urol.

